# A good-for-something second brain

**DOI:** 10.1038/s41467-023-44472-9

**Published:** 2024-01-03

## Abstract

As part of our tenth anniversary celebrations, the editorial team at *Nature Communications* wanted to hear from early career researchers who have published with us. We asked the early career researchers to tell us in an essay what is amazing about the research question(s) that drove them and the highs—and lows—of the journey from hypothesis to publication.

**Figure:**
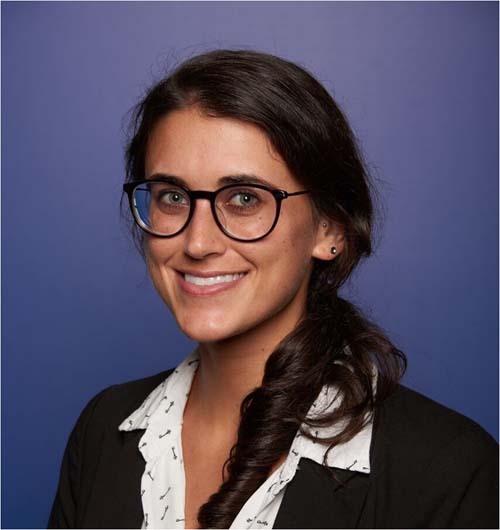
Rachael Hachadorian (Dr Hachadorian’s photo was taken by Michael Woodward - AAPM Deputy Executive Director)

Rachael Hachadorian is currently a fourth-year PhD Candidate at Dartmouth College in the Thayer School of Engineering for Principal Investigator Professor Brian Pogue. Her research is focused on monitoring of cancer patients receiving radiation by imaging the Cherenkov light released during dose delivery, in real-time. Specifically, she is working on quantifying field match, and minimizing inter-patient differences which limit the linearity that exists between Cherenkov light and deposited radiation dose. There is a patent pending for techniques she has implemented.

As an early career researcher and an author of a paper published by *Nature Communications*^1^, tell us about the journey you’ve been on?

*Nature Communications* published my work earlier this year, and hardly anything had been more academically gratifying. I think as most researchers can attest to, we interface on-and-off with a touch of imposter syndrome in our early careers. So, to finally see something that came from my own devising published in a high-impact journal, offered me assurance that I had made the right decision to stay a researcher, and that (at least some of) my ideas were worth reading about.

The vast majority of my research motivation comes from a gratitude, almost a debt, that I feel I owe to medical doctors. It gives me the opportunity to give back to a community that has provided me with so much help, so I was thrilled to begin patient studies with the physicians in the Radiation Oncology department at Dartmouth-Hitchcock. The idea that my research could potentially, one day, provide them with real-time dose maps to verify each day of their prescribed treatment was both rewarding and motivating.

I am a 28-year-old PhD student, and grateful for all the things that I am able to do: I have backpacked in the Sierra Nevada mountains, played college softball, and chased kids down a mountain as a volunteer snowboard coach. Usually, these are the types of things that healthy, mobile individuals do, but for me it wasn’t always the case.

I was participating in an NSF REU physics internship in 2012. I crouched under a computer to check a cable connection and another physicist joked that it looked like a second brain was growing out of the side of my knee. Quite unfortunately, it was not a second brain, which could have at least maybe been useful brainpower. A month later I was diagnosed with a systemic autoimmune disorder, treated the same way that rheumatoid arthritis is.

Being in the middle of my busiest college semester, the two physicians who took over my care accommodated my insane schedule into theirs. Over multiple synovial aspirations, two steroid injections, two surgeries, four medication changes and eight colds (but who’s counting), they allowed me to essentially have them on speed dial. My doctor put me on a new medication and found a way for me to afford it. I soon had my mobility back and finally was not getting sick all the time.

Becoming a medical doctor may have been a logical pursuit at that point, but I always loved physics, space, and how things worked. So, I thought that perhaps I could be helpful to physicians or a greater medical cause if I could offer a perspective different than that of another medical doctor. So far, this has turned out to be rewardingly true. We have helped each other to see and understand things that the other did not or might not have considered. Thus, often, I find myself looking for ways to see clinical scenarios as they do, then use my own background to analyze them. In ways, this is what led me to this study, which sought to develop Cherenkov images as a surrogate for absorbed dose from CT scans.

One weeknight in early April, I sat on my colleague’s couch with our computers running and coffee brewing, getting ready for another late night of being snowed in. Having spent the day debugging, I was frustrated, foggy, and had to actively remind myself to think from a relevant clinical perspective. When that’s the case, I often revert back to a default questions of ‘what resources do we have access to? What can we use?’ I opened up CT scans knowing at least every patient would have one. I toggled through slices in Image-J and took ROI’s of various areas on the breast. I reconsidered that Hounsfield units (HU) are related to the electron density, or the mean free path. Essentially, that is how far the photon will travel probabilistically before it is absorbed or scattered. It would make sense to utilize this information that inherently must exist for each patient’s treatment plan. The adipose to fibroglandular ratios seem pretty qualitatively disparate, so it really begged the question: Could a strong-enough correlation exist between Cherenkov light and breast HU to devise a correction methodology around?

You have likely guessed by now that there was. Coefficients of variation describing macroscopic tissue properties that once spread from 20 to 30% now spread from 7 to 13%. There are several other important factors that make Cherenkov light nonlinear to dose, but this was the largest piece of the puzzle.

This publication (Hachadorian et al. *Nature Communication* 11, 2298 (2020)) was co-authored by a radiation oncologist, a medical physicist, two professors of engineering, and a software engineer. Together, we make up an exceptionally diverse research and patient care team, each having our own strengths. I am thrilled to be part of it, and I am eager to see where this research takes us.

I am grateful that even though that second brain couldn’t contribute any brainpower, it in some ways led me here. Doctors, clinicians, and researchers everywhere deserve to be applauded. They’ve inspired new researchers such as myself, and they have made it possible to increase a patient’s quality of life. That, to me, is quite invaluable.
